# Immunonutritional consequences of different serine-type protease inhibitors in a C57BL/6 hepatocarcinoma model

**DOI:** 10.18632/oncotarget.26605

**Published:** 2019-01-22

**Authors:** Jose Laparra, Bartosz Fotschki, Claudia Haros

**Affiliations:** ^1^ Madrid Institute for Advanced studies in Food (IMDEA Food), Ctra, 28049 Madrid, Spain; ^2^ Department of Biological Function of Food, Institute of Animal Reproduction and Food Research, Polish Academy of Sciences, 10-748 Olsztyn, Poland; ^3^ Instituto de Agroquímica y Tecnología de Alimentos (IATA), Consejo Superior de Investigaciones Científicas (CSIC), Parque Científico, Paterna, 46980 Valencia, Spain

**Keywords:** hepatocarcinoma, serine-type protease inhibitors, macrophage, innate immunity, immunonutrition

## Abstract

Imbalances in innate immunity and the activity of innate immune cells are implicated in the development of hepatocellular carcinoma (HCC). Plant seeds are good sources of protease inhibitors, which can have a significant influence on human health disorders, especially in the field of cancer prevention. To elucidate the impact and preventive effects of immunonutritional serine-type protease inhibitors (STPIs) on HCC, it was used an established model of chemically induced liver injury. Injured livers induced Akt as well as hepatic infiltration of NKG2D^**+**^ and CD74^**+**^ cells. Feeding STPIs reduced size and number of intrahepatic nodes of mononuclear. These animals showed an inverse association of the severity of HCC with bioactive hepcidin levels, which was significantly correlated with the hepatic myeloperoxidase activity. According to their origin, administration of STPIs significantly induce increased numbers of F4/80^**+**^ cells in injured livers that can be responsible for the biological effects detected on the parenchyma and inflammatory markers under DEN/TAA treatment. These findings can have direct implications in HCC immunotherapy where enhanced response(s) in inflammation-driven cancer patients could help promoting inflammation-driven processes and favor tumor growth. Altogether, this study demonstrates that oral administration of STPIs modulate innate immunity response influencing HCC aggressiveness and progression. These results represent a path forward to develop durable, long-lasting response against hepatocarcinoma and open a future research path in the development of coadjutant intervention strategies to pharmacological therapies.

## INTRODUCTION

Hepatocellular carcinoma (HCC) is the most common primary liver neoplasm and the third leading cause of cancer-related death worldwide with more than 850,000 cases diagnosed each year [1]. The development of this pathology is closely linked to dietary habits that favor hepatic immunometabolic alterations that give rise to hepatic steatosis with low grade inflammation (NAFLD), a common link to pathologies such as obesity and type 2 diabetes [2]. Most HCC cases are detected in a stage of advanced progression, which leads to a poor prognosis and highlights the need for a preventive/therapeutic intervention in the disease.

Immunological disbalances reported in HCC are highly relevant for the prognosis and definition of an immunotherapeutic treatment [3]). In this scenario, the idea that immune-oncological ‘efficacy’ can be enhanced if immunotherapy is combined with a strategy that improves the activation processes of antitumor immune response(s) is gaining wide acceptance [4]. Recent research has provided new insights into how innate and adaptive lymphocytes operate sequentially to establish tissue metabolic homeostasis [5]. Here authors identified a key role for innate lymphoid cells and intestinal epithelial cells to shape lipid homeostasis. An abnormal lipid handling contributes to excessive accumulation favoring lipotoxicity and cell dysfunction, recognized risk factors for the development of NAFLD and HCC progression. There is established evidence that appropriate changes in lifestyle, particularly diet, can prevent one third of all cancers for which is a major determining factor for its development [6]. This has led to the consideration that composition of the diet, rather than its caloric intake and its interaction with the intestinal microbiota, as well as the immune system is critical in the establishment and progression of inflammatory processes in the ‘gut-liver axis’.

The scarce models to study HCC due to the lack reproducibility of the immunometabolic complexity of the tumor microenvironment in the *in vivo* situation, hinders, to a certain extent, the advances in the treatment of the disease. This has been partially overcome by the identification of genetic signaling systems that govern hepatocellular carcinogenesis through genomic studies, which can be corroborated in several murine models [7, 8]. In relation to these genetic findings, the mouse model of HCC induced by the procarcinogen diethylnitrosamine (DEN), has been shown to reflect an etiologically comparable course with HCC in humans [7]. HCC progression in chemically injured livers appears dependent on the intestinal microbiota, and TLR4 activation in non-bone marrow-derived resident liver cells [9]. TLR4 activity in tumor-recruited immune cells promotes immunosuppressive effects by modifying secreted cytokines in the tumor microenvironment and T-cell maturation [10]. Interventions targeting TLR4 have in fact been proposed as with significant potential for treating cancer. However, TLR4 signaling is also important for induction of antitumor response [11]). Moreover, TLR4 signaling is critical to drive an oriented macrophage polarization towards an M1 phenotype and synergize with interferon-γ to render macrophages tumoricidal [12].

Plants are good sources of serine-type protease inhibitors (STPIs) [13]. So far, crops such as beans, potatoes, barley, squash, millet, wheat, buckwheat, groundnut, chickpea, pigeonpea, corn, and pineapple have been identified as good sources of STPIs. Despite the widespread presence of STPIs in different grains their potential to stimulate and the effects on innate immune response(s) are variable [14]. Notably, STPIs have also been examined as therapeutic agents, primarily to deal with various human cancers [14]. Most of current research has focused on the intestinal inflammatory potential of wheat STPIs [15, 16] establishing the engagement of the TLR4-MD2-CD14 complex [15]. Otherwise, the relatively high homology between STPIs from different origin and their potential effect on immunonutritional processes that may influence TLR4 signaling and the tumor environment have been studied to a much lesser extent.

The presence of STPIs in grains displaying a potential capacity as inducers of innate immune responses may results critical for successful control of the tumor microenvironment as well as priming of adaptive immunity. Thereby, STPIs could contribute to prevent HCC progression. Thus, this study aims to explore the immunonutritional consequences of STPIs in a chemically-induced murine HCC model, and on the possible role of STPIs contributing to control HCC aggressiveness and liver injury.

## RESULTS AND DISCUSSION

### Liver injury in DEN/TAA-treated mice

**Figure 1:**
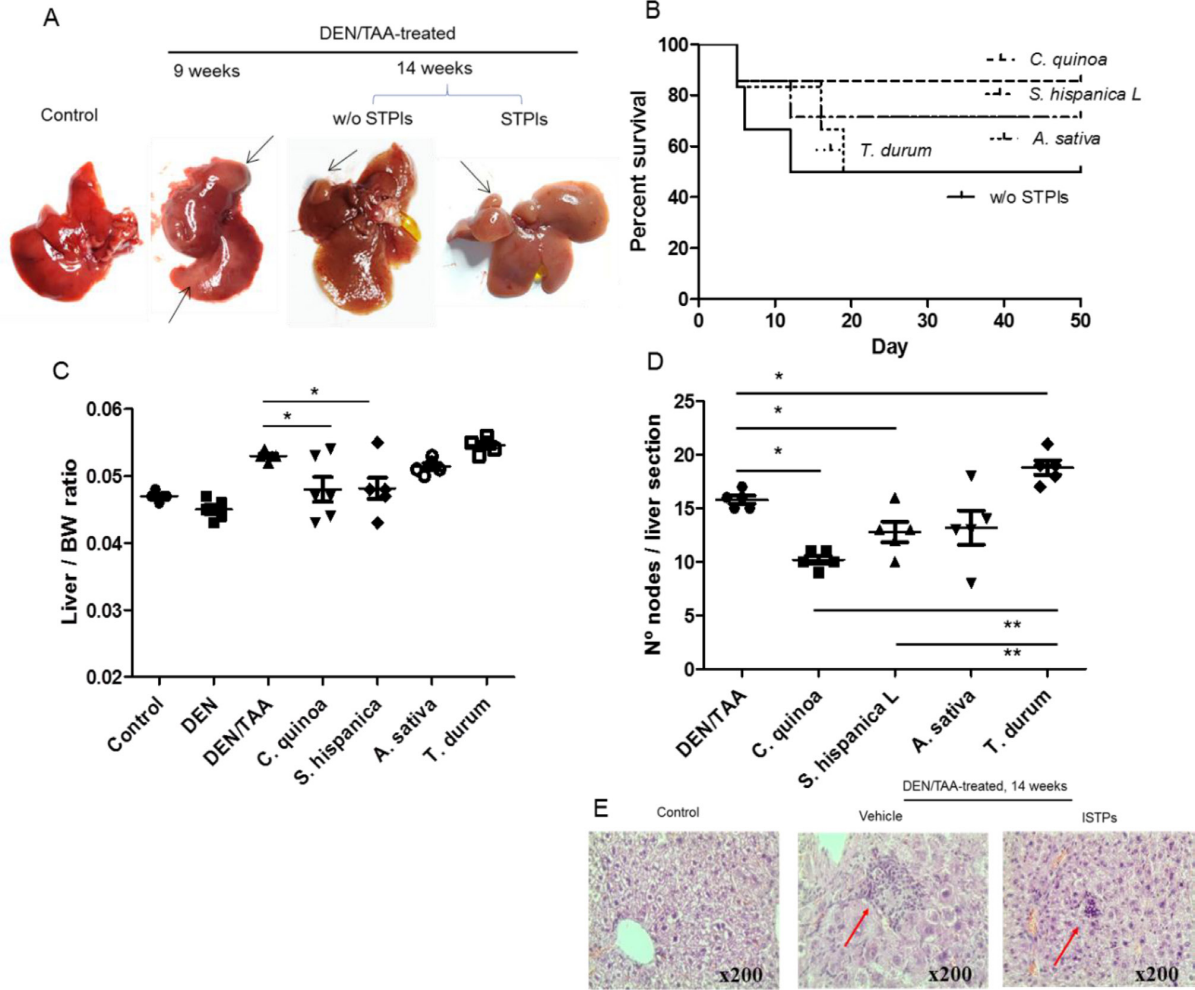
Co-administration of diethylnitrosamine (DEN) and the hepatotoxin thioacetamide (TAA) promotes liver tumor development in mice (**A**) Gross images of C57BL/6 wild-type mouse livers at 14 weeks of age (eight weeks post DEN/TAA treatment). Arrows indicate liver discoloration after 9 weeks of DEN administration and the HCC development after 14 weeks in animals administered or not (w/o) with STPIs from *C. quinoa*. (**B**) Survival curve of serine-type protease inhibitors (STPIs) administered mice. (**C**) Liver to body weight (BW) ratio of DEN/TAA-treated mice fed with STPIs. (**D**) Incidence of intrahepatic nodes of mononuclear cells in animals DEN/TAA-treated mice developing HCC and administered with STPIs from *C. quinoa*. (**E**) Hematoxylin and eosin staining (×200) of wild-type mouse livers at 14 weeks of age in control (untreated) animals and DEN/TAA-treated mice administered with STPIs obtained from *C. quinoa* in comparison to those not receiving STPIs. ^*^Indicates statistical differences (Tukey–Kramer’s test, *P* < 0.05) to DEN/TAA-treated animals not receiving STPIs.

Major features in the development of cancer-linked to chronic inflammation were assessed in the model of chemically-induced hepatocellular carcinoma (HCC) (Figure 1). Repeated doses of the mutagenic and carcinogenic DEN were administered to 6 weeks old male mice together with the oral administration of the hepatotoxin TAA inducing the development of HCC for 8 weeks [9] (Figure 1A). Cohorts of DEN- and DEN/TAA-treated mice and untreated age-match control mice were assessed over time for illness and survival. It was observed a greater mortality in animals subjected to the concurrent administration of DEN and TAA (Figure 1B), wherein 50% of DEN/TAA versus 100% of DEN-treated mice completed the 8 weeks of treatment. The small HCC development after DEN/TAA treatment that is due to the relatively short study period points out a ‘fulminant hepatitis’ as most likely cause of deaths rather than cirrhosis or HCC. In addition, of the mice that reached the treatment (14 weeks of age), DEN/TAA-treated mice displayed a significantly higher liver/body weight ratio than those receiving DEN alone (Figure 1C). Notably, animals fed with the extracts enriched in serine-type protease inhibitors (STPIs) displayed lower mortality as well as liver/body weight ratio compared to those not receiving the extracts. The grade of liver lesions was assessed according to the number of intrahepatic nodules of mononuclear cells via histopathology (Figure 1D, 1E). In animals fed with STPIs the intrahepatic nodules accounted with lower incidence levels than those not receiving the extracts during HCC development. This phenotype confirms the decreased tumor burden (intrahepatic nodules size and liver/body weight ratio) caused by DEN/TAA in mice that did not receive the extracts. The results clearly revealed significant differences in relation to the protective effects derived from the STPIs according to their origin: C. quinoa > *S. hispanica* L, *A. sativa* > *T. durum*.

**Figure 2:**
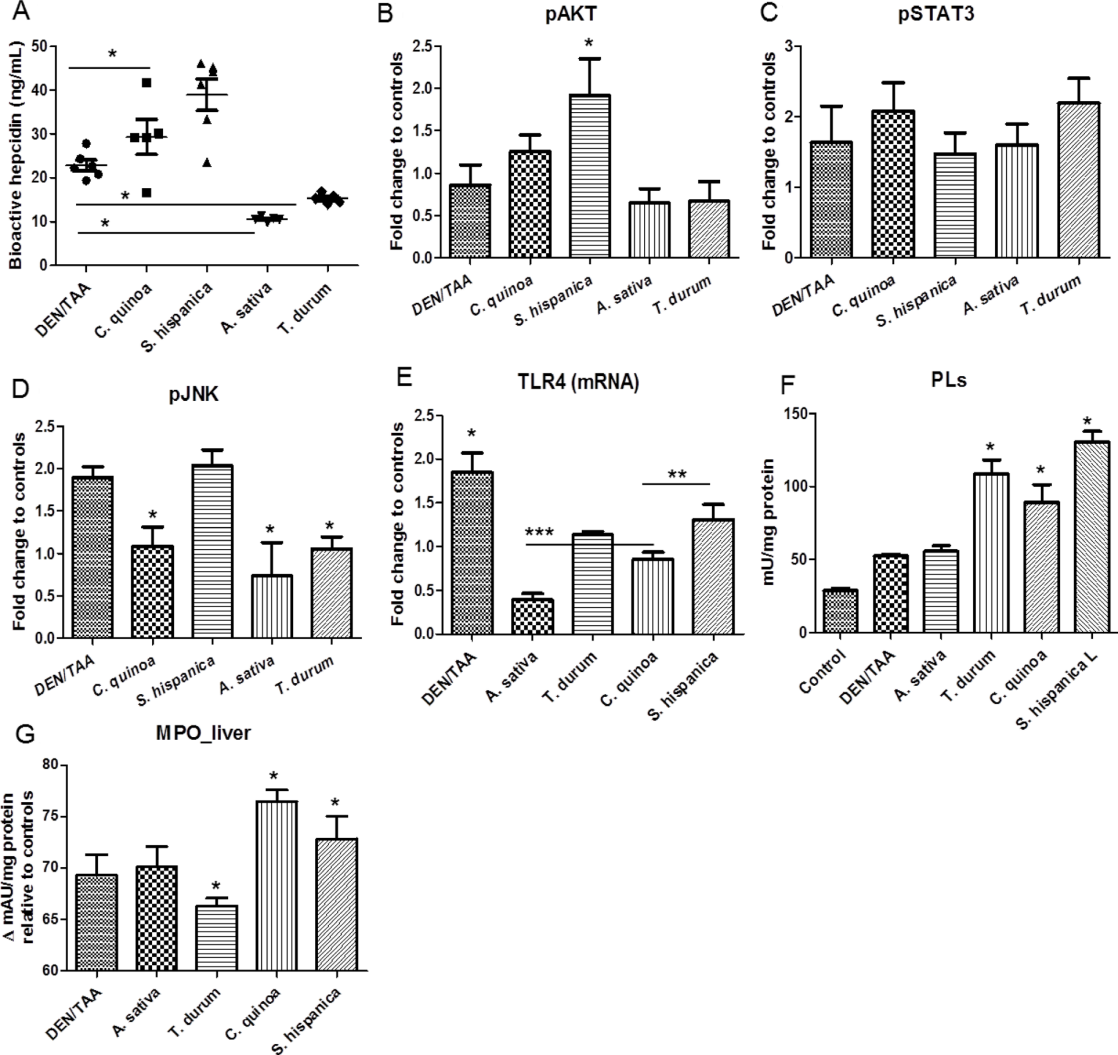
Immunonutritional changes in C57BL/6 wild-type mouse livers at 14 weeks of age (eight weeks post DEN/TAA treatment) administered with serine-type protease inhibitors (STPIs) administered mice (**A**) Influence of STPIs in bioactive hepcidin production. (**B**–**D**) Levels of activation of AKT, STAT3 and JNK in mouse livers. Four to six samples per each group were used for the Western blot analysis, and representative images are shown. Tubulin was used as loading controls. (**E**) Hepatic expression (mRNA) levels of TLR4 in DEN/TAA treated animals after administration of STPIs. (**F**) Hepatic total phospholipid content and changes in myeloperoxidase activity (**G**). Results are expressed as mean ± SEM (4–6). ^*^Indicates statistical differences (Tukey–Kramer’s test, *P* < 0.05) to DEN/TAA-treated animals not receiving STPIs.

The production of bioactive hepcidin in the development of cancer-linked to chronic inflammation was quantified in the model of chemically-induced HCC (Figure 2). Animals administered with DEN/TAA displayed significantly suppressed (by 14%) mean hepcidin levels (Figure 2A) in relation to untreated control animals (24.5 ng/mL). This effect seems to correspond to the development of human HCC where gene expression analyses on tissues showed that *HAMP* was transcriptionally repressed in HCC tissues [17]. Hepatocyte growth factor, among other, has been suggested as a potential regulator of hepcidin suppression in chronic liver diseases [18]. Bioactive hepcidin levels were found significantly decreased also in animals fed with STPIs from *A. sativa* and *T. durum* Besides, administration of STPIs from *C. quinoa* and *S. hispanica* L increased bioactive hepcidin levels (up to 1.7-fold) relative to DEN/TAA-treated animals not receiving STPIs. Since hepcidin suppresses liver fibrosis by impeding ferroportin mediated regulation of AKT [19], it is hypothesized that increased hepcidin levels help reducing tumor progression in severity. When comparing the numbers of intrahepatic nodes in the groups of ‘low’ or ‘high’ hepcidin levels, plasmatic hepcidin concentrations showed a multiplicative relationship [ln (intrahepatic nodules) = 3,486–0,293*ln(hamp); *p* = 0.04] to the means counts for intrahepatic nodules. Collectively, these results show that immunonutritional STPIs have a substantial positive effect on tumorigenesis, reducing the progression of HCC in DEN/TAA-treated mice, without influencing the type of tumors that ultimately develop.

When comparing hepcidin production in different animal models of chemically-induced HCC, it was confirmed that mice treated with CCl_4_ for 6 weeks, hepcidin protein levels were also decreased in the liver [19]. In the analysis of human data, hepcidin levels have been reported to be downregulated in patients as the disease worsens, supporting that the loss of hepcidin plays a role in the progression of HCC [20]. In addition, systemic hepcidin has also been found to increase and correlate with T-stage of colorectal cancer [17] and metastasis and IL-6 in renal and prostate cancer [21, 22].

Hepcidin suppression, which is observed in liver stress conditions where HCC develops [17, 23], is linked to mitogen stimulation and hepatic growth factors via components of Ras/RAFMAPK and mTOR signaling [18]. Here, to elucidate the molecular mechanisms underlying hepatocarcinogenesis in DEN/TAA-treated mice, it was assessed the activation of AKT/mTOR and Ras/MAPK pathways in these mice by Western blotting (Figure 2B). Levels of activated/phosphorylated AKT (S473) were increased in liver lesions from DEN/TAA-treated mice and lower than in mice fed with STPIs from *S. hispanica* L. Besides, expression levels of p-AKT in DEN/TAA-treated mice resulted significantly higher than in those receiving STPIs from *A. sativa* and *T. durum*. The expression of STAT3, a well-studied activator of hepcidin transcription required only for IL-6 driven induction of hepcidin, was lowest in animals fed with *S. hispanica* L and maximum in those administered with STPIs from *T. durum*. Activation of the Ras/MAPK cascade, as indicated by pJNK levels, seems to be suppressed in DEN/TAA-treated animals receiving STPIs from *C. quinoa*, *S. hispanica* L or *A. sativa*. JNK signaling pathway is linked to the STAT3 activation [24]. In line with this, the lower JNK expression levels in relation to those of STAT3 seem to suggest this possibility as a likely scenario. The implication of JNK in STAT3 activation contributes to growth-arrest and apoptosis protecting from DEN/TAA-induced cytotoxicity. Altogether, these results indicate that activation of AKT/mTOR and Ras/MAPK cascades is a molecular feature of DEN/TAA driven hepatocarcinogenesis. However, STPIs from *C. quinoa* or *S. hispanica* L seem to display crucial roles preventing mTOR activation as supported by plasmatic hepcidin concentrations.

Hepcidin expression has been suggested to be regulated by TLR4 expressed by hepatocytes via the IRAK-TRAF6-JNK-AP-1 axis [25]. As TLR4 agonists (i.e., LPS) and defined phospholipids (PLs) activate some of the same intracellular signaling pathways and genes, for example MCP-1 and IL-8, there were quantified the total intrahepatic PLs concentrations in the different groups of treatment (Figure 2C–2D). Animals administered with extracts from *T. durum* or *C. quinoa* had comparable PLs concentrations and higher than those animals that did not receive STPIs or from *A. sativa* (Figure 2F). Meanwhile, while those fed with *S. hispanica L* exhibited highest PLs values. These results support the suggested differences in the breadth of TLR4 signaling as PLs flippases (P4-ATPases) attenuate TLR4 signaling by mediating endocytic retrieval of the receptor [26]. The latter study suggested the involvement of PLs flippases in innate immune response mediating the internalization of TLR4. Thus, changes in the PLs concentration could contribute to different sensitivities within the IL-6/STAT3 axis and lead to a disparity in downstream TLR4 signaling as reflected in bioactive hepcidin levels.

### Innate immunity in injured liver

**Figure 3:**
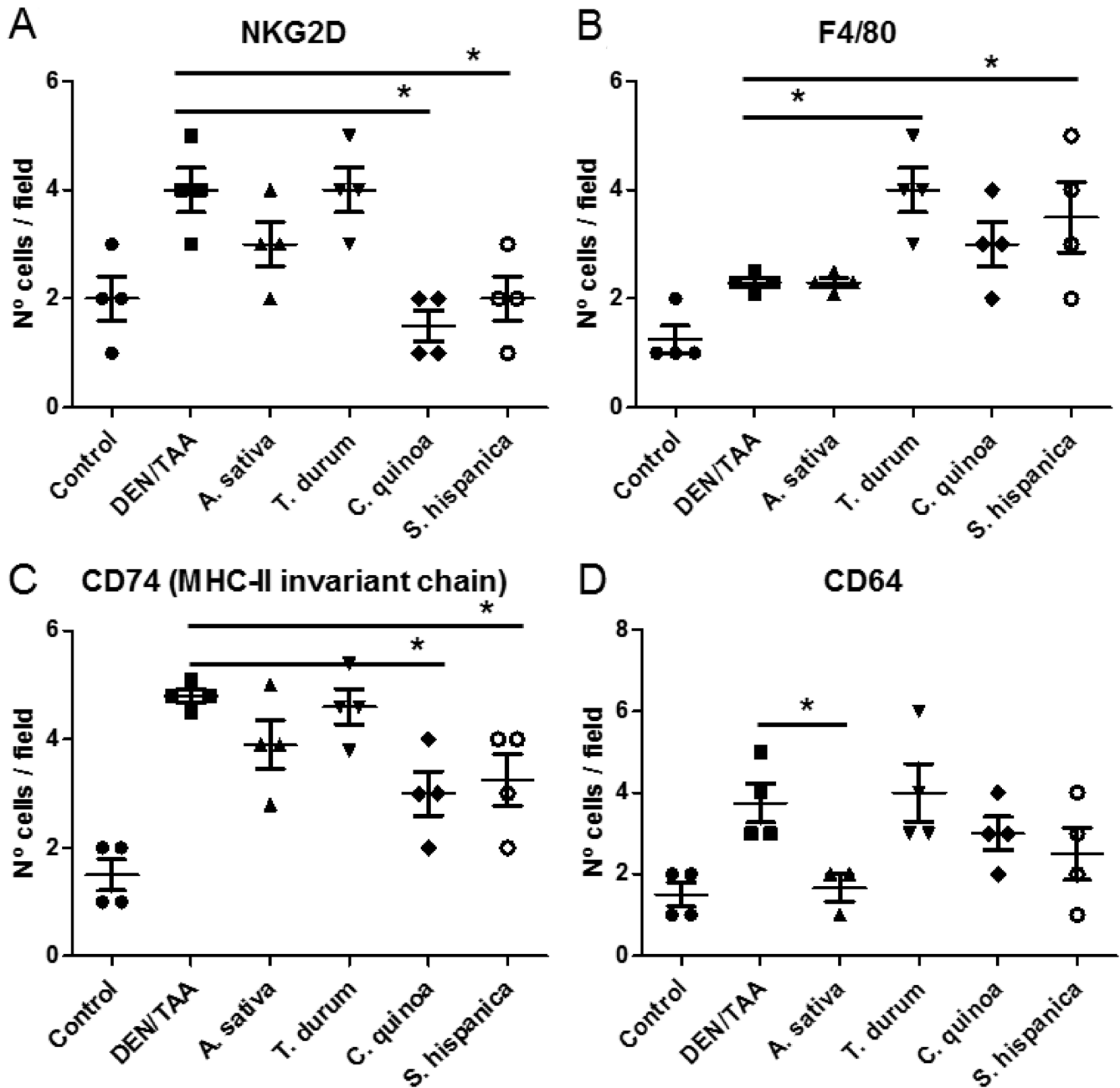
Immunological changes in C57BL/6 wild-type mouse livers at 14 weeks of age (eight weeks post DEN/TAA treatment) administered with serine-type protease inhibitors (STPIs) administered mice Number of hepatic single cells stained for NKG2D (**A**), F4/80 (**B**), CD74 (MHC-II invariant chain) (**C**) and CD64 (**D**) quantified by immunofluorescence analysis. Cell counting was performed in 5 to 10 well orientated microscopic fields from each 3 stained sections per group. Results are expressed as mean ± SEM (4–6). ^*^Indicates statistical differences (Tukey–Kramer’s test, *P* < 0.05) to DEN/TAA-treated animals not receiving STPIs.

Tumor-infiltrating immune cells proportions during HCC development are highly relevant for disease prognosis and effectivity of immunotherapy [3]. Here, there were identified some of the immune populations infiltrating injured livers of DEN/TAA-treated mice that can play a key role improving or worsening HCC severity (Figure 3). When comparing with healthy livers from non-treated age matched mice, immunohistochemical (IHC) analyses evidenced a significant enrichment in NKG2D^+^, CD74^+^, F4/80^+^ and CD64^+^ cells, in tumor bearing mice. Particularly, increased numbers of NKG2D^+^ cells (Figure 3A) are concordant with the positive role of the NKG2D-ligand pathway promoting tumor growth in DEN-treated mice [27]. The latter study reported a NKG2D-dependent CD8^+^ (memory) T cell enrichment accumulated in the surrounding tissue of tumor bearing mice. JNK/STAT3 signaling, linked to the sustained activation of the tyrosine kinase Lck by CD28 activation in CD8^+^ T cells, increases NKG2D expression [28]. Enhanced anti-tumor immunity requires the interplay between resident and circulating memory CD8^+^ T cells [29]. Also, it is known the inhibitory impact of DEN on the inhibitor of nuclear factor kappa-B kinase (IKK-β), which is reflected in higher numbers of the CD74^+^ population (Figure 3B), favoring and increased the hepatic susceptibility to chemical damage and development of HCC [30]. Only animals fed with STPIs from *C. quinoa* and *S. hispanica* L showed significantly lower number of intrahepatic CD74^+^ cells than DEN/TAA-treated animals that not received STPIs.

An increased expression of CD74 is closely associated with the production of the macrophage migration inhibitor factor [31]. It was further distinguished the immunonutritional impact of STPIs administration on the intrahepatic macrophage population identifying the anti-tumoral M1 phenotype based on the F4/80 glycoprotein [32, 33] (Figure 3C). The number of F4/80^+^ cells was slightly higher in animals DEN/TAA-treated animals not receiving STPIs compared con untreated controls. Notably, except for the administration of STPIs from A. sativa all other groups of treatment exhibited a 2-fold higher intrahepatic F4/80^+^ population. The macrophage F4/80 receptor is required for the induction of antigen-specific efferent regulatory T cells in peripheral tolerance [34]. Thus, overexpression of the F4/80 glycoprotein can help preventing autoimmune reactivity against non-cancerous cells from the DEN/TAA-injured livers. This increase in the F4/80 populations is concordant with the JNK driven STAT3 activation (Figure 2B) as IL-6/STAT3 signaling pathway is inhibited in M1-type macrophages [35].

The expression of CD64 (neutrophil Fcγ receptor I) may have a role in the evaluation of therapy as it changes with the course of the disease [36]. Thus, serving as a biomarker to evaluate the condition of DEN/TAA-induced injury and the ameliorative effect of STPIs (Figure 3D). The expression of CD64 was associated to the lymphocyte population based on changes in the macrophage/monocyte as well as granulocyte population. The results showed that administration of STPIs from *C. quinoa* and *S. hispanica* L ameliorated DEN/TAA-induced liver injury. Taken together, these data suggested that inducible F4/80^+^ cells in animals administered with *C. quinoa* and *S. hispanica* L exerted an antitumor function but not immunosuppressive effects. Further, we found in pace with the hypothesized immunosuppressive effect, the level of CD64^+^ cells also increased significantly associated to F4/80^+^ in animals administered with STPIs from *T. durum*.

**Figure 4:**
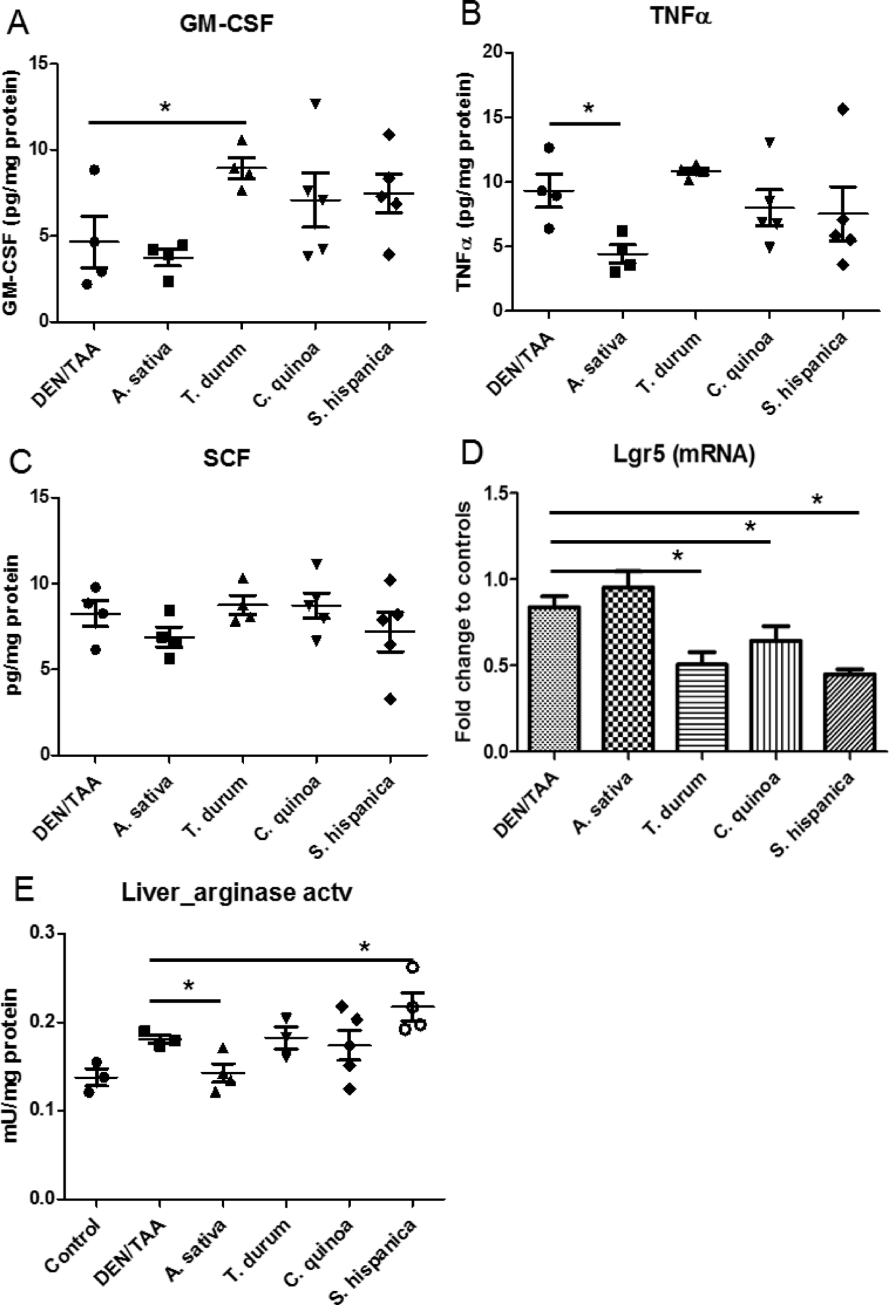
Hepatic concentration of immunological mediators in C57BL/6 wild-type mouse livers at 14 weeks of age (8 weeks post DEN/TAA treatment) administered with serine-type protease inhibitors (STPIs) administered mice Tissue cytokine concentration: (**A**) granulocyte-monocyte colony stimulating factor (GM-CSF), (**B**) Tumor necrosis factor (TNF)-α, (**C**) Stem cell factor (SCF). (**D**) Expression (mRNA) levels of Lgr5 gene. (**E**) Changes in liver arginase activity in animals fed with STPIs. Results are expressed as mean ± SEM (4–6). ^*^Indicates statistical differences (Tukey–Kramer’s test, *P* < 0.05) to DEN/TAA-treated animals not receiving STPIs.

Immune mediators such as GM-CSF and TNFα that are known to favor the recruitment of immune cells to the damaged liver were remarkably decreased in DEN/TAA-treated animals in comparison with untreated controls (Figure 4A–4B). These observations could reflect alterations and dysfunction of the hepatic parenchyma following tissue injury that seems to affect immune control. The results showed that GM-CSF induced the proliferation of F4/80^+^ cells. TNFα concentrations did not change in parallel to the GM-CSF production. In animals administered with STPIs, levels of hepatic SCF were at similar levels than in DEN/TAA-treated mice that not received none the extracts (Figure 4C). Whereas, only the administration of extracts from *A. sativa* kept the expression (mRNA) levels of Lgr5 at values similar to those found in control animals (Figure 4D). Collectively, these results illustrate that the concurrent administration of DEN/TAA contributes significantly to liver injury affecting immune dysfunction and the administration of STPIs from *C. quinoa* and *S. hispanica* help to overcome these effects at a significant extent protecting the hepatic function.

GM-CSF facilitates the recruitment and expansion of macrophages, neutrophils as well as myeloid derived suppressor cells in several HCC models [37]. However, its immunological effects depend to a higher extent on the contextual environment. The significant positive correlation of GM-CSF with F4/80^+^ cells (*r* = 0,685; *r*^2^ = 46,9%; *p* = 0,005) can echo the increase of activated macrophages during the pathophysiologic inflammatory response [38]. It cannot be concluded whether increased numbers of F4/80^+^ cells in animals administered with the extracts is associated with increased phagocytic function. However, decreased phagocytic activity with decreased number of intrahepatic nodules of mononuclear cells (Figure 1D) may interpret the experimental data showing immunosuppressive effects on persistent cycles of liver injury, inflammation, and hepatocyte proliferation, which aggravate HCC progression. In this study, hepatic TNFα concentrations did not parallel the expression of arginase activity (Figure 4E), which is required for tumor associated macrophages to induce immunosuppressive effects and apoptosis of T cells [39].

### Neutrophils in DEN/TAA-induced injury

**Figure 5:**
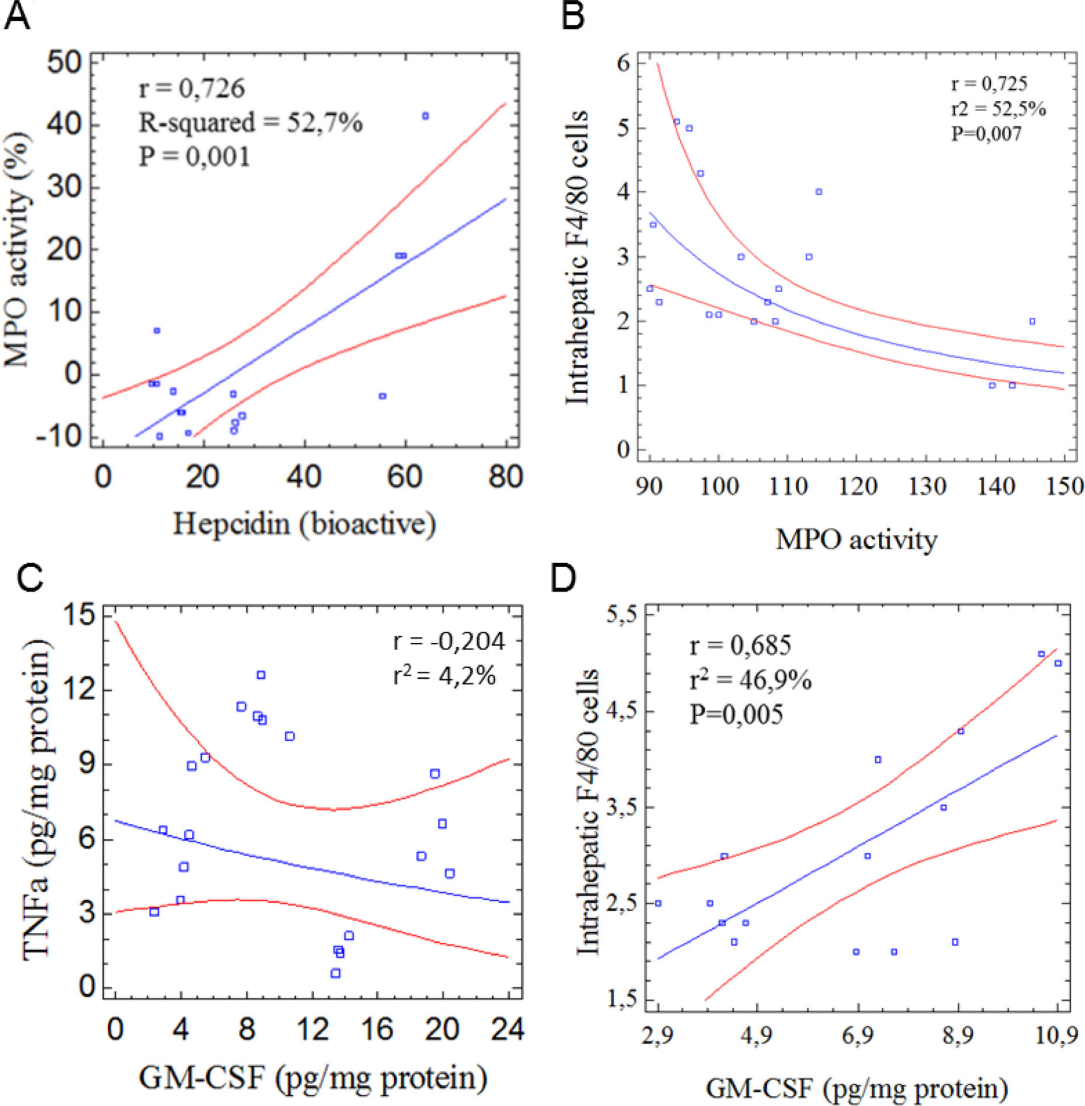
Statistical evaluation of the potential role of neutrophils in liver tumor development in mice treated with diethylnitrosamine/thioacetamide (**A**) Intrahepatic myeloperoxidase activity (MPO) to plasmatic bioactive hepcidin concentrations; (**B**) MPO to intrahepatic F4/80^+^ cells; (**C**) Association between TNFα and GM-CSF to elucidate tumor-induced PD-L1 activation; (**D**) Intrahepatic F4/80+ cells to GM-CSF. Correlations were calculated using the Statgraphics v.5.1 (Enterprised Ed.).

Neutrophils (NTs) have been identified as an unexpected component of the immune system contributing to hepcidin production [40]. It appears that NTs either inhibit or promote tumor progression by immunosuppressive effects on innate and adaptive cells. To approach the response of NTs and activation in tumor microenvironment it was quantified the myeloperoxidase activity (MPO) in DEN/TAA-injured livers (Figure 5). Intrahepatic MPO activity (Figure 2G) was positively correlated with hepcidin (Figure 5A). However, MPO was inversely correlated to intrahepatic F4/80+ cells (Figure 5B). The immunosuppressive effect of NTs on adaptive immunity has been found to be mediated by the tumor cell-derived GM-CSF production, which efficiently induces PD-L1 on NTs through activation of JNK/STAT3 signaling. Notably, no correlation between intrahepatic MPO activity and GM-CSF, nor between GM-CSF and TNFα (Figure 5C–5D), which cooperates with GM-CSF to induce PD-L1, was quantified. The correlation found between MPO and hepcidin could be consistent with a role for NTs in resolving or dampening inflammatory processes and, thereby, ameliorating DEN/TAA-induced liver injury. This is in line with the phospholipids and α-1 antitrypsin factor reduced NTs degranulation [41] and the impact of TLR4 signaling on MPO losing the potency to activate neutrophil degranulation [42] (Figure 2C–2D).

Neutrophils can mimic myeloid-derived suppressor cells (MDSCs) and mediate immunosuppression depending on local conditions [43, 44]. Consequently, the distinction of these two cell populations is a matter of relevant importance to obtain a detailed understanding of the potential contribution of NTs to HCC progression. MDSCs (CD45^+^CD33^low^CD11b^dim^) have shown to morphologically resemble NTs and contribute to CD8^+^ T cell suppression via the IL-6/IL-8-arginase I axis (PI3K-AKT signaling pathway) [44]. In this study, intrahepatic MPO activity (Figure 2E) did not correlate with arginase activity (Figure 4E). Besides, MPO activity positively associated with overall HCC decreased severity. The increase in MPO activity in animals fed with STPIs from *C. quinoa* and *S. hispanica* L may partially explain a decreased proportion of MDSCs, while reducing tumor formation [45].

Otherwise, Gr-1^+^CD11b^+^F4/80^+^ cells from tumor-bearing mice do not suppress, but activate NK cells to produce high amounts of IFNγ [46]. The increased production of IFNγ could synergize with STPIs from *C. quinoa* and *S. hispanica* L, which influence the retrieval of TLR4, reducing the apoptosis-inducing capacity of Gr-1^+^Cd11b^+^F4/80^+^ cells [11]. This defined phenotype (Gr-1^+^CD11b^+^F4/80^+^) was shown to express the retinoic acid early inducible gene 1 (RAE-1) for the activating receptor NKG2D. RAE-1 proteins are found on activated, but not resting, macrophages and have been found to down-regulate NKG2D expression on NK cells during innate immune response(s) to be mediated by Toll-like receptor signaling through the MyD88 adaptor molecule [47]. The lower intrahepatic nodes (Figure 1D) in animals administered with from *C. quinoa* and *S. hispanica* L displaying a decreased hepatic expression of NKG2D is concordant with a role for NKG2D in sustaining liver damage and death of hepatocyte [27]. The lack of significant correlation between intrahepatic MPO activity and F4/80^+^ cells also helps discarding the tumor associated neutrophils recruitment of immunosuppressive macrophages to promote HCC progression [48]. Altogether, these results point to a positive effect of intrahepatic NTs contributing to reduce tumor progression and increasing immune cell activation during tumor progression.

In summary, animals administered with DEN that additionally receive TAA develop innate and adaptive immune responses resembling early stages where HCC develops. The disruption of hepatic immunity as well as metabolic homeostasis in the chemically-induced HCC model appears to play a fundamental role in the burden of liver injury and onset of the disease. These results are in line with recent studies showing immune imbalances that contribute to maintain an innate inflammatory environment and ultimately tumor growth. Administration of STPIs significantly induce an activation and increased numbers of F4/80^+^ cells in injured livers that can be responsible for the biological effects detected on the parenchyma and inflammatory markers under DEN/TAA treatment. The differences observed in immune response(s) according to STPIs administration reveal their different immunogenic potential and immunonutritional behavior for the activation antitumor responses. These findings can have direct implications in HCC immunotherapy where enhanced response(s) in inflammation-driven cancer patients could help promoting inflammation-driven processes and favor tumor growth.

As targeted immunological therapies grow in complexity, understanding and controlling immune regulation is of greater importance in the design of new therapies to reduce the risk of developing HCC and to develop more effective treatments. Here, the effectivity of STPIs from *C. quinoa* and *S. hispanica* L improving the quality of antitumoral immune response(s) on HCC prevention can contribute to tailor preventive and/or therapeutic interventions for large groups of populations at risk. However, because of the relevance for health and social impact of cancer, additional research is needed to confirm and adequately quantify the impact of STPIs in HCC progression/aggressiveness by human trials.

## MATERIALS AND METHODS

### Sample preparation

Commercial samples (*Triticum durum* flour, *Avena sativa* flour and seeds from *Chenopodium quinoa* and *Salvia hispanica* L) were finely milled and homogeneously grinded. The obtained flours were extracted with a phosphate buffered saline solution (PBS, 137.0 mM NaCl, 2.7 mM KCl, 8.1 mM Na_2_HPO_4_, 1.5 mM KH_2_PO_4_, pH 7.2-7.4) [16]. Briefly, grounded aliquots (0.5 g) were weighed into a centrifuge tube (50 mL) and extracted (×2) with 5 mL of PBS at 37° C with gentle agitation during 2 h. Afterwards, the suspensions were centrifuged for 15 min at 4000 × g. The corresponding soluble fractions were pooled, heated (60° C/30 min) and centrifuged (8000 × g). The supernatants were filtered through a 0.45 µm membrane. Total soluble protein concentrations in the extracts were determined using a Lowry method based commercial kit (TP0200, Sigma) and lyophilized.

### Mice, HCC induction and evaluation

Male mice were obtained from the Centro de Investigaciones Biológicas (CIB-CSIC) (Madrid, Spain). Animal experiments were carried out in strict accordance with the recommendations in the Guide for the Care and Use of Laboratory Animals of CSIC (Consejo Superior de Investigaciones Científicas) and the protocol was approved by its Ethic Committee and the regional government. Before inducing the hepatocarcinoma, animals were randomly distributed into nine different groups (*n* = 6 per group): 1) received vehicle (control); 2–5) received extracts enriched in SETIs from the first DEN injection; 6–9) received extracts enriched in SETIs from the week before first DEN injection. In mice (C57Bl/6), HCC was induced by the combination of DEN (20 mg/kg, i.p.) given at age of 6-week-old, and 24 weekly (3-times per week) administrations of TAA (saturated solution −0.5 ml/kg i.g., dissolved in PBS - dose equivalent to 80 mg/kg). Mice were sacrificed 8 weeks after the initial DEN injection.

Changes in body weight and food consumption were monitored every two days. After treatment, mice were sacrificed by cervical luxation. Whole blood samples were preserved in EDTA treated tubes (at room temperature) for analyses. Sections (1 cm) of the liver were fixed in 4% paraformaldehyde, embedded in OCT (Thermo Scientific™), immersed in RNA later buffer (Qiagen, USA), Krebs’s buffer or RIPA buffer and kept at −80° C until analysis.

### Histologic and morphometric evaluation

Liver sections (5 μm) were stained with haematoxylin-eosin staining. The samples were analysed with a Nikon Eclipse 90i microscope equipped with a Nikon DS-5Mc digital camera. Photos were analysed with the Nis Elements software (Nikon Instruments Inc., Melville, USA). The parameters analysed included, number of intrahepatic nodules of mononuclear cells and disorganization of the hepatic parenchyma.

### Immunofluorescence analysis

Liver (4 μm) sections were incubated with a blocking solution (1X PBS/5% normal serum/0.3% Triton™ X-100) for 60 min. After blocking, samples were washed with PBS. Adequate fluorescent-tagged antibodies (NKG2D, CD64, CD74 and F4/80) (Biolegend) were diluted in the dilution buffer (1X PBS/1% BSA/0.3% Triton™ X-100) and pipetted onto the slides. Samples were incubated overnight. Subsequently, after 3 washes of 10 min each in PBS-Triton X-100 0.05% (v/v) liver sections were analyzed using an inverted fluorescence microscope Leica DM IL LED.

### Quantification of bioactive hepcidin

All sample preparation steps were performed at room temperature as previously described [49]. Aliquots (50 μL) of plasma were pipetted into 200 μL cone tubes and a 100 μL aliquot of acetonitrile (Burdick and Jackson, Muskegon, MI, USA) was added to each tube and mixed by pipetting. The samples were then centrifuged at 3000 g for 10 min at 4° C (Jouan, Winchester, VA, USA). After centrifugation, the protein precipitation supernatant (100 μL) was mixed with 0.02% (v/v) aqueous acetic acid and injected on an Agilent HPLC system. The column used in these analyses was a Poroshell C18 5 μm 2.7 × 50 mm (Agilent).

### Cytokine protein and phospholipid assay

Liver sections kept in Krebs’s buffer (1 ml), containing the Complete Protease Inhibitor Cocktail (Sigma), were thaw up and homogenized using a TissueRuptor (Qiagen) freshly before use. Then, samples were centrifuged (10,000 ×g/15 min) to get clear supernatants for cytokine determination. Tumor necrosis factor (TNF)-α and granulocyte-monocyte colony stimulating factor (GM-CSF) (ImmunoTools) were determined by ELISAs according to the manufacturer’s instructions. Different aliquots of liver sections were homogenized prior to quantification of the total phospholipids content using a commercial ELISA kit (Abcam). The results of the ELISA assays were normalized according to the total protein content (Pierce^®^-BCA, Thermo Scientific™).

### Quantitative reverse transcription real-time polymerase chain reaction (qRT-PCR)

Validated Gene Expression Assays for murine TLR4 (forward 5′-TGG GAA CAC ACG GTT GGA AA -3′, reverse 5′-ACA GCA AGT TGT AGC ACT ACT GA-3′), CD36 (forward 5′-AAA AGC CAA GCC AGT GAC AAG-3′, reverse 5′-GGA CGC TTT TTC CTC AAG TCA-3′), CD68 (forward 5′-AGA AGT GCA ATG GTG GGT CT-3′, reverse 5′-TGG GGC TTA AAG AGG GCA AG -3′), Lgr5 (forward 5′- GGA CCA GAT GCG ATA CCG C-3′, reverse 5′- CAG AGG CGA TGT AGG AGA CTG -3′) and β-Actin (forward 5′-TGC ACC AAC TGC TTA-3′, reverse 5′-GGA TGC AGG GAT GAT GTT-3′) were purchased from Applied Biosystems (Foqter City, CA). PCR reactions were performed with 500 ng of cDNA from liver sections, using the Universal PCR Master Mix (Applied Biosystems). Cycling conditions were: 10 min of denaturation at 95° C and 40 cycles at 95° C for 15 s and at 65° C for 30 s and 72° C for 20 s. Quantitative values were calculated by using the 2^−ΔCt^ method, wherein the ΔCt value of each sample was calculated by subtracting the average Ct value of the target gene from the average Ct value of the GAPDH gene.

### Protein extraction and western blotting

Frozen liver samples were homogenized in RIPA buffer, containing the Complete Protease Inhibitor Cocktail (Sigma), using a TissueRuptor (Qiagen) and sonicated. Protein concentrations were determined with the Pierce^®^-BCA Protein Assay Kit (Thermo Scientific™) using bovine serum albumin as standard. Aliquots (40 μg) of the lysate were denatured by boiling in Tris-Glycine SDS Sample Buffer (Invitrogen), separated by SDS-PAGE, and then transferred onto nitrocellulose membranes (Invitrogen, Grand Island, NY). Membranes were blocked in 5% non-fat dry milk in Tris-buffered saline containing 0.1% Tween 20 for 1 hour and probed with specific antibodies (BD Pharmingen) – pAKt (S472/S473) (Cat. No. 550747), pSTAT3 (S727) (Cat. No. 612542) and pJNK (T183/Y185) (Cat. No. 612540. Each primary antibody was followed by incubation with secondary antibody diluted 1:10,000 for 1 hour and then revealed with the SuperSignal West Pico Chemiluminescent Substrate (Pierce Chemical Co., New York, NY). Equal loading was assessed by tubulin Western blotting (Cat. No. 55632).

### Measurement of myeloperoxidase (MPO) activity

MPO activity was measured in the supernatants of hepatic tissue homogenates as a marker for neutrophil infiltration. Aliquots of supernatants (50 μl) were assayed in a reaction mixture that contained 110 μl PBS, 20 μl of 0.22 M NaH_2_PO_4_ (pH 5.4), 20 μl of 0.026% (v/v) H_2_O_2_, and 20 μl of 18 mM tetramethylbenzidine in 8% (v/v) aqueous dimethylformamide. After 10 min of reaction at 37° C, 30 μl sodium acetate (1.5M; pH 3) was added, and the absorbance at 620 nm was read in a microtiter plate reader. The activity was expressed as mU/mg protein.

### Statistical analyses

Statistical analyses were performed using SPSS v.15 software (SPSS Inc., Chicago, IL, USA). For normally distributed data ANOVA and the Student *t* test were applied and, for non-normally distributed data, the Mann–Whitney *U* test was used. Statistical significance was established at *P* < 0.05 for all comparisons.
